# Hypnotism and Drunkenness

**Published:** 1893-01-28

**Authors:** 


### HYPNOTISM AND DRUNKENNESS.

IN a pamphlet published by Messrs. Churchill, Dr. Charles Lloyd Tuckey deals with the results of treating chronic alcoholism by hypnotism. The results he chronicles are, on the whole, encouraging; but they are hardly such as to make anyone regard hypnotism as a specific for drunkenness. In the first place hypnotism demands the patient's consent, sharing this limitation with all other remedies. Now, a very large number of drunkards have no wish to be saved from the demon of drink; they hate the consequences, the headache and nausea; they are annoyed at the loss of business and the like, which their habit entails, but drinking seems to them a pleasant thing in itself, and they do not care to lose the pleasure. This at once limits the number to whom hypnotism is helpful; for Dr. Tuckey is careful to point out that the patient must help the operator by sharing in the desire for cure, and puts himself in a passive state only that this desire may be more firmly impressed upon his brain. The moral nature is not to be weakened by submission to a stronger will, but developed by the suggestions of the hypnotic state. But this makes it difficult to see where the method of hypnosis comes in. We can see the value of passivity for subjugation and mere impression; but when active co-operation is needed it seems more reasonable to ask it from a fully conscious subject. Dr. Tuckey condemns retreats for inebriates, as doing nothing to strengthen the moral fibre of the inmates, and seems to think the mere absence of temptation leaves the drunkard as prone to fall as before. Of course the idea of the retreat is that of physical cure, the elimination of alcohol from the organism, and the restoration of the cells which presumably are injured. The moral surroundings are healthful, but not exactly bracing; the inmates tend to become, as one of them has said, "incurable loafers." They are never tempted, nor do they ever need to exert themselves; thus they leave the retreat no stronger morally than when they entered. Dr. Tuckey claims, on the other hand, that hypnotism developes the will; but as his treatment, too, demands continued supervision and repeated experiments, it runs in the lines of all other cures, though it claims to require a briefer space of time. In dealing with such a fatal taint as drunkenness any cure is worth trying; but Dr. Tuckey's pamphlet cannot be regarded as justifying more than moderate expectations from hypnotism as a cure; and, indeed, he claims no more.

